# 

**Published:** 1843-09

**Authors:** 


### AMERICAN JOURNAL

OF

### Dental Science.

PUBLISHED UNDER THE AUSPICES OF THE

### American Society of Dental Surgeons.

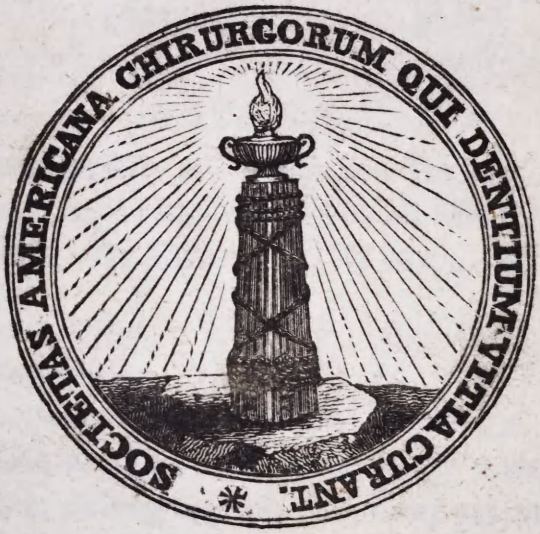

VOLUME IV.—1843-4.

EDITORS:

CHAPIN A. HARRIS, M. D.
SOLYMAN BROWN, M.D.
EDWARD MAYNARD, M. D.

SURGEON GEN'L'S OFFICE LIBRARY.

NEW YORK:—GEORGE ADLARD,
BALTIMORE:—ARMSTRONG & BERRY,
LONDON:—SAMUEL HIGHLEY.

1844.

W
1
AM 45 M

WOODS & CRANE, PRINTERS,
*Baltimore.*

